# Daily sedentary time and physical activity as assessed by accelerometry and their correlates in older adults

**DOI:** 10.1186/s11556-019-0210-9

**Published:** 2019-02-18

**Authors:** Adriana J. van Ballegooijen, Hidde P. van der Ploeg, Marjolein Visser

**Affiliations:** 1 Department of Health Sciences, Faculty of Science, Amsterdam Public Health Research Institute, Vrije Universiteit, De Boelelaan 1085, 1081 HV Amsterdam, the Netherlands; 2 Department of Public and Occupational Health, Amsterdam Public Health Research Institute, VU University Medical Center Amsterdam, Amsterdam, the Netherlands

## Abstract

**Background:**

Higher physical activity is associated with lower chronic disease risk among older adults. However, less is known about the optimal balance between daily physical activity and sedentary time and their correlates among older adults. We described objectively measured physical activity patterns using 7 day hip-accelerometry and assessed its correlates in a large cross-sectional sample of the Longitudinal Aging Study Amsterdam, a population-based cohort of older Dutch adults. In addition, we examined different combined profiles of sedentary time and physical activity across strata of sex, age, education and BMI groups.

**Results:**

Mean age was 71 (SD 8) years and 51% (*n* = 615) were women. The majority of wear time was spent sedentary (65%) followed by light (33%), and MVPA (2%). Higher age and higher BMI were related to more time spent sedentary, while female sex and lower education were related lower sedentary time. The combination of high sedentary time (≥65.4% of waking time) and low physical activity (< 9.1% of waking time) was significantly associated with higher age, higher BMI, and slower walking speed compared to the combination of low sedentary time and high physical activity *P* < 0.001.

**Conclusions:**

Dutch older adults spend on average 65% of their waking time sedentary. Older adults’ sedentary time differs by age, sex, education and BMI groups. The combination of high sedentary time and low physical was associated with higher age, higher BMI, and slower walking speed compared to the combination of low sedentary time and high MVPA. This suggests that increasing light activity might be an effective and feasible strategy in older persons to reduce sedentary time. Future studies should assess whether low- sedentary and high-light physical activity are associated with improved long-term health outcomes (also independent of MVPA).

**Electronic supplementary material:**

The online version of this article (10.1186/s11556-019-0210-9) contains supplementary material, which is available to authorized users.

## Introduction

Physical activity in old age is a key factor in maintaining physical functioning and to reduce the risk of age-related diseases [1–4]. Previous studies have shown that the amount of moderate to vigorous intensity physical activity (MVPA) is lowest in old age compared to other age groups as measured by hip-accelerometry, [5] while the amount of sedentary time is highest in old age as measured by a questionnaire for sedentary behaviors and MVPA [6].

The majority of the physical activity evidence has been measured by self-report, while less is known about objectively measured physical activity [7]. Self-reported data are prone to reporting errors and recall bias, and is mainly focused on MVPA [8–10]. Therefore, there is a clear need for more objective physical activity data to assess lower physical activity intensities.

During the last decade, there has been a growing interest in the health effects of sedentary behavior and the optimal balance between daily sedentary and physical activity behaviors [11, 12]. Next to the health benefits of MVPA, accumulating evidence suggests that light-intensity physical activity is related to numerous health benefits such as improved obesity markers, glucose control and survival [13, 14]. This would suggest that inactive adults should be encouraged to reduce sedentary behavior and engage in any intensity of physical activity.

Identifying joint associations of sedentary time and physical activity (light, moderate, and vigorous) and their correlates is essential in order to promote healthy behaviors and develop more effective guidelines [15]. More evidence is needed on how sedentary time and physical activity are interrelated and distributed across age categories and other demographic and health factors [16–20]. Therefore, the purpose of this study was to comprehensively describe objectively measured sedentary and physical activity patterns and to assess its correlates in a large population-based sample of older persons. In addition, we examined joint combinations of different sedentary time and physical activity profiles across strata of sex, age, education and BMI groups.

## Results

Of the 1201 LASA participants, mean age was 70.7 (SD 8.0) years (range of 58–99 year) and 615 (51%) were women (Table 1). The majority of the participants were low educated, were non-smokers, lived in rural areas and shared a household. Mean BMI was 22.9 (SD 3.8) kg/m^2^, self-rated health was generally good/excellent (72%) and over the last 2 weeks (70%) performed bicycling, and 9% performed swimming activities. Women were lower educated, more often living alone, underweight, had more functional limitations, slower walking speed, and lower self-rated health than men (*P* < 0.01).

**Table 1:** Characteristics of 1201 LASA participants of 7 day hip-accelerometer study

	Total group	Men	Women
Demographic factors			
N	1201	586 (49%)	615 (51%)
Mean age	70.7 ± 8.0	70.5 ± 7.7	70.9 ± 8.2
Age categories			
< 65 years	317 (26%)	153 (26%)	164 (27%)
≥ 65–70 years	330 (28%)	172 (29%)	158 (26%)
≥ 70–75 years	218 (18%)	109 (19%)	109 (18%)
≥ 75–80 years	165 (14%)	76 (13%)	89 (15%)
≥ 80 years	171 (14%)	76 (13%)	95 (15%)
Education			
Low	389 (32%)	170 (29%)	219 (36%)
Middle	461 (38%)	196 (33%)	265 (43%)
High	351 (29%)	220 (38%)	131 (21%)
Urbanization grade			
Rural	540 (45%)	252 (43%)	288 (47%)
Intermediate urban	436 (36%)	231 (39%)	205 (33%)
Urban	224 (19%)	103 (18%)	121 (20%)
Living situation			
Alone	342 (29%)	106 (18%)	236 (38%)
Together	859 (71%)	480 (82%)	379 (62%)
Season			
Summer	246 (21%)	117 (20%)	129 (21%)
Fall	294 (24%)	147 (25%)	147 (24%
Winter	347 (29%)	174 (30%)	173 (28%)
Spring	314 (26%)	148 (25%)	166 (27%)
Lifestyle factors			
Smoking			
Current	130 (11%)	61 (11%)	69 (11%)
Former	724 (61%)	392 (68%)	332 (55%)
Never	326 (28%)	123 (21%)	203 (34%)
BMI categories			
Underweight^a^	187 (16%)	37 (7%)	150 (25%)
Normal	690 (59%)	356 (62%)	334 (56%)
Overweight	241 (20%)	151 (26%)	90 (15%)
Obese	54 (5%)	29 (5%)	25 (4%)
Health and function factors			
Number of chronic diseases			
0	253 (21%)	143 (24%)	110 (18%)
1	354 (30%)	181 (31%)	173 (28%)
≥ 2	594 (49%)	262 (45%)	332 (54%)
Self-rated health			
Excellent/good	862 (72%)	449 (77%)	413 (67%)
Poor/fair	338 (28%)	137 (23%)	201 (33%)
≥ 1 functional limitation	421 (35%)	174 (30%)	247 (40%)
6 m walk test (sec)	7.2 ± 3.4	7.00 ± 2.8	7.5 ± 3.8
Bicycling	835 (70%)	420 (72%)	415 (68%)
Swimming	108 (9%)	39 (7%)	69 (11%)
Accelerometry results			
Wear time (h/d)	14.2 ± 1.5	14.2854 ± 1.5	14.1 ± 1.5
Sedentary time (h/d)	9.2 ± 1.5 (65%)	9.4 ± 1.4 (66.5%)	9.0 ± 1.4 (64.1%)
Light-low time (h/d)	3.5 ± 1.0 (25%)	3.3 ± 0.9 (23.2%)	3.7 ± 1.0 (26.4%)
Light-high time (h/d)	1.1 ± 0.7 (7.6%)	1.1 ± 0.7 (7.5%)	1.1 ± 0.7 (7.6%)
Moderate time (min/d)	14 (5–28) (2.3%)	18 (7–32) (2.7%)	11 (4–23) (1.9%)
Vigorous time (min/d)	0 (0–0) (0.0%)	0 (0–0) (0.0%)	0 (0–0) (0.0%)

Mean ± standard deviation median and interquartile range or number and percentage;

For accelerometry results: mean time ± SD, or median (interquartile range) plus % out of total wear time in brackets

^a^BMI underweight: < 70 years < 18.5 kg/m^2^, ≥ 70 years < 20 kg/m^2^

h/d: hours per day, min/d: minutes per day

The non-participating persons (*N* = 194) were slightly older 71.8 vs 70.7 years and more often women 54 vs 51%, and had a higher BMI 24.8 vs 22.9 kg/m^2^ than participating persons (*N* = 1218).

### Physical activity intensities

Mean accelerometer wear time was 849 min per day, or approximately 14 h per day. On average, participants spent 9.2 h (551 min) of total wear time sedentary (65%), and 4.6 h (277 min) in light (33%) and 14 min in moderate physical activity (2%). Vigorous activity was only performed by 108 participants with a range between 1 and 47 min per day.

**Fig. 1:**
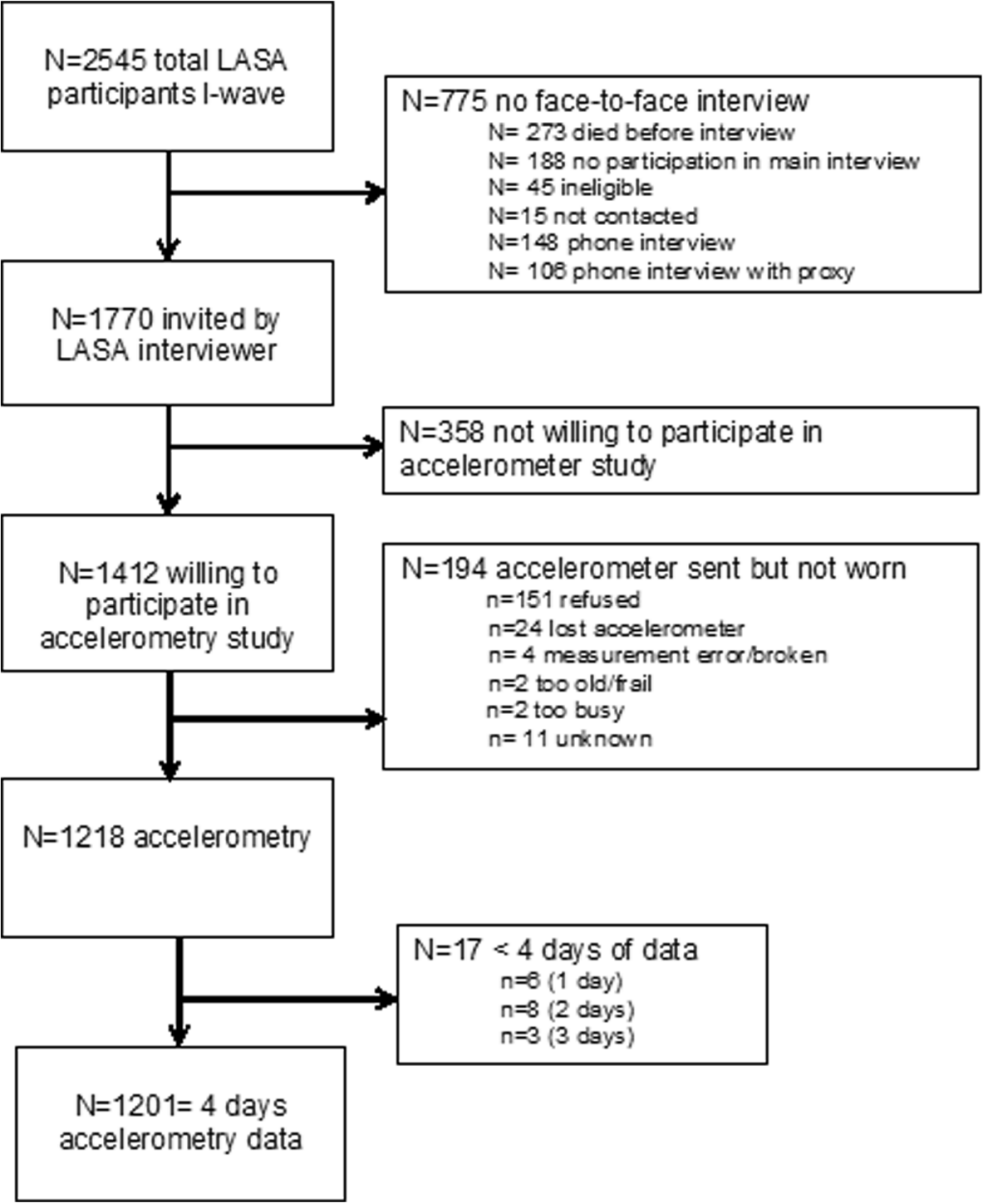
Flow diagram of LASA participants for 7 day hip-accelerometer study

The percentage of sedentary time was inversely (linearly) related with both the intensity categories light-high (≥ 760 cpm) and vigorous MVPA (≥2020) expressed as percentage of wear time (Fig. 1). Sedentary time was strongly correlated with the intensity categories light low and light high r = − 0.81 and r = − 0.85, respectively, while sedentary time was only modestly correlated with the moderate intensity physical activity r = − 0.48, P-for all < 0.01.

### Sedentary time and physical activity according to age, sex, education and BMI

Higher age was significantly associated with less wear time, with less time spent on light low, light high and MVPA, and with more time spent sedentary expressed as percentage of total wear time (Table 2, *P* < 0.001). Female sex was associated with less time spent sedentary and MVPA, and with more time in the light-low and light-high intensity categories. Lower education was associated with less time sedentary and MVPA, and with more time spent in light-low and light-high intensity activities. Higher BMI categories were related to more time sedentary and in light-high intensity activities, and less time in light-low and MVPA.

**Table 2:** 7-day hip-accelerometry results^a^ by age, sex, education and body mass index groups in 1201 LASA participants

	Wear time (min)	Sedentary (%)	Light-low (%)	Light-high (%)	MVPA (%)
Age categories					
<65 years	877 (867-886)	63.2 (62.2-64.1)	26.0 (25.4-26.6)	8.5 (8.1-8.9)	2.8 (2.6-3.0)
65-70 years	850 (841-859)	64.5 (63.7-65.4)	25.3 (24.7-25.9)	7.8 (7.4-8.2)	2.7 (2.5-2.9)
70-75 years	852 (841-863)	64.5 (63.5-65.6)	25.5 (24.7-26.2)	7.7 (7.2-8.2)	2.1 (1.8-2.3)
75-80 years	838 (825-850)	65.7 (65.5-66.9)	24.4 (23.6-25.3)	7.5 (6.9-8.1 )	2.0 (1.7-2.3)
≥80 years	807 (794-820)	71.4 (70.1-72.6)	21.3 (20.4-22.2)	5.0 (4.4-5.6)	1.7 (1.4-2.0)
*P*-trend	<0.001	<0.001	<0.001	<0.001	<0.001
Sex					
Men	853 (846-860)	67.1 (66.4-67.8)	23.4 (23.0-23.9)	7.1 (6.7-7.4)	2.9 (2.7-3.0)
Women	847 (840-854)	63.6 (62.9-64.3)	26.1 (25.7-26.6)	7.9 (7.6-8.2)	1.9 (1.7-2.0)
*P*-trend	0.260	<0.001	<0.001	<0.001	<0.001
Education					
Low	845 (837-853)	63.8 (63.0-64.6)	25.6 (25.0-26.1)	8.2 (7.8-8.6)	2.1 (2.2-2.3)
Middle	847 (839-854)	65.3 (64.6-66.0)	24.9 (24.4-25.4)	7.4 (7.1-7.8)	2.1 (2.2-2.4)
High	858 (849-867)	66.9 (66.0-67.8)	23.9 (23.3-24.5)	6.8 (6.4-7.2)	2.9 (2.7-3.1)
*P*-trend	0.073	<0.001	<0.001	<0.001	<0.001
BMI categories					
Underweight^b^	853 (840-865)	64.9 (64.7-66.1)	25.8 (25.0-26.7)	6.8 (6.3-7.4)	2.7 (2.4-3.0)
Normal	854 (848-860)	64.7 (64.1-65.3)	25.3 (24.9-25.7)	7.7 (7.4-7.9)	2.4 (2.3-2.6)
Overweight	840 (829-850)	66.4 (65.3-67.4)	23.6 (22.9-24.3)	7.6 (7.1-8.1)	2.0 (1.7-2.2)
Obese	828 (805-849)	69.3 (67.2-71.5)	21.1 (19.7-22.6)	7.2 (6.2-8.2)	1.9 (1.3-2.4)
*P*-trend	0.033	<0.001	<0.001	0.069	<0.001

Analyzed with analysis of covariance with 95% confidence intervals. Significant *P* < 0.05 when no overlap occurs in 95% confidence interval between categories. ^a^Adjusted for all other variables listed in table plus MVPA, except for MVPA, which was adjusted for all variables listed plus sedentary time. ^b^BMI underweight: < 70 years < 18.5 kg/m^2^, ≥ 70 years < 20 kg/m^2^

### Correlates of sedentary time and physical activity

The demographic factors age, sex, education and season accounted for 16.6% of the variance in sedentary time (R^2^ = 0.166 *P* < 0.001) (Table 3). The lifestyle factors smoking and higher BMI categories contributed to more time sedentary, while high urbanization was related to less time sedentary, which explained more of the total variance (R^2^ = 0.207 *P* < 0.001). A higher walk test time, functional limitations as well as a poor/fair self-rated health were related to more time sedentary, while bicycling was related to lower sedentary time (final model R^2^ = 0.288, *P* < 0.001).

**Table 3:** Hierarchical regression analysis of correlates of sedentary time^a^ in 1201 LASA participants

	Block 1			Block 2			Block 3		
	β	SE	*P*-value	β	SE	*P*-value	β	SE	*P*-value
*Demographic factors*									
Age ≤ 65 years (ref)	0.0			0.0			0.0		
Age 65–70 years	1.6	0.7	0.019	1.9	0.7	0.006	1.5	0.7	0.029
Age 70–75 years	2.4	0.8	0.004	2.8	0.8	0.001	2.0	0.8	0.004
Age 75–80 years	4.2	0.9	< 0.001	4.5	0.9	< 0.001	3.7	0.8	< 0.001
Age ≥ 80 years	11.4	0.9	< 0.001	12.0	0.9	< 0.001	8.9	0.9	< 0.001
Men (ref)	0.0			0.0			0.0		
Women	−2.5	0.5	< 0.001	−2.0	0.5	< 0.001	−2.6	0.5	< 0.001
Education low	−1.8	0.7	0.007	−2.4	0.7	0.001	−2.4	0.6	< 0.001
Education medium	−0.3	0.7	0.658	−0.7	0.6	0.268	−1.3	0.6	0.036
Education high (ref)	0.0			0.0			0.0		
Spring (ref)	0.0			0.0			0.0		
Summer	−0.5	0.8	0.519	−0.4	0.8	0.608	−0.6	0.7	0.410
Autumn	1.3	0.7	0.073	1.6	0.8	0.031	1.6	0.7	0.025
Winter	2.4	0.7	0.001	2.4	0.7	0.001	2.3	0.7	0.001
*Lifestyle factors*									
Never smoker (ref)				0.0			0.0		
Former smoker				−0.5	0.6	0.367	−0.4	0.6	0.446
Current smoker				2.6	0.9	0.006	1.8	0.9	0.042
BMI category underweight				−0.6	0.8	0.440	0.2	0.7	0.840
BMI category normal weight (ref)				0.0			0.0		
BMI category overweight				8.0	2.6	0.002	6.6	2.4	0.008
BMI category obese				8.5	1.6	< 0.001	6.3	1.6	< 0.001
Urbanization low (ref)				0.0			0.0		
Urbanization intermediate				−0.2	0.5	0.703	0.1	0.5	0.888
Urban high				−5.0	2.5	0.046	−4.3	2.4	0.072
*Health and function factors*									
Duration 6 m walk test (s)							0.5	0.1	< 0.001
No functional limitation (ref)							0.0		
≥ l functional limitation							1.7	0.6	0.008
Bicycling (yes)							−0.9	0.2	< 0.001
Self-rated health poor/fair							2.6	0.6	< 0.001
Self-rated health good/excellent (ref)							0.0		
Explained variance (R^2^)	0.166		< 0.001	0.207		< 0.001	0.288		< 0.001

^a^Expressed as percentage of total wear time. Ref: Reference. Lower values imply less time spent in sedentary

Unstandardized regression coefficients and standard errors (SE)

Consecutive blocks of correlates:

Block 1: age, sex, education and season;

Block 2 block 1 and smoking, BMI categories and urbanisation;

Block 3: block 2 and 6 m walk test, functional limitations, bicycling, and self-rated health

Living situation and number of chronic diseases were not significant correlates

The correlates of physical activity (≥ 760 cpm light-high intensity and higher) were mostly in the opposite direction compared to sedentary time. A lower amount of its variance was explained by the demographic, lifestyle and health measures (R^2^ 0.176, 0.203, and 0.258, respectively, P < 0.001, as compared to sedentary time. The correlates of MVPA were in line with light-high physical activity, (Additional file 1: Tables S1–S2).

Combined sedentary time and physical activity patterns (based on the sample median) indicated that men were more often high sedentary and high physically active (72%) compared to women (28%), while women were more often low sedentary and low physically active (70%) compared to men (30%) (Table 4). The high sedentary and low physical activity group was significantly older and had a higher BMI compared to the other groups. The high sedentary and high physical activity group had the fastest 6 m walk test. The low sedentary and low physical activity group spent relatively the highest percentage of waking time in light-low activity. The combined sedentary and MVPA patterns were slightly more pronounced.

**Table 4:** Adjusted means across four combined sedentary time and (light-high to vigorous intensity^a^) physical activity profiles in 1201 LASA participants

	High sedentary Low PA	High sedentary High PA	Low sedentary Low PA	Low sedentary High PA	*P*-value
N	*N* = 490	*N* = 110	N = 110	*N* = 491	
Men	244 (50%)	79 (72%)	33 (30%)	230 (47%)	
Women	246 (50%)	31 (28%)	77 (70%)	261 (53%)	
Adjusted means					
Age (years)	72.4 (71.7–73.1)^b^	69.1 (67.7–70.5)	72.2 (70.7–73.6)	68.9 (68.2–69.5)	< 0.001
BMI (kg/m^2^)	23.4 (23.0–23.7)	22.9 (22.2–23.6)	22.9 (22.2–23.7)	22.5 (22.1–23.7)	0.017
6 m walk test (sec)	7.4 (7.1–77)	6.3 (5.7–6.9)	7.7 (7.1–8.3)	7.1 (6.8–7.4)	0.002
Light-low (%)	21.8 (21.4–22.2)	20.6 (19.8–21.4)	30.9 (30.1–31.7)	27.5 (27.1–27.9)	< 0.001

*PA* physical activity, adjusted means: estimated with analysis of covariance

^a^≥760 cpm which includes light-high, moderate and vigorous intensity activity, expressed as percentage of total wear time

Categorization based on median: sedentary < 100 (cpm) < 65.4/≥65.4% and Light high ≥760 (cpm) < 9.1/≥9.1% out of total wear time

^b^ANCOVA: analysis of covariance with 95% confidence intervals. Significant *P* < 0.05 when no overlap occurs in 95% confidence interval between categories

Adjusted for sex, education categories, age, BMI and 6 m walk test

### Sensitivity analysis

Excluding participants with potentially biased data due to lower wear time or a break in wear time did not substantially change the results (Additional file 1: Tables S3–S8).

## Discussion

This study described objectively measured sedentary time and physical activity and their correlates in a large population-based sample of Dutch older adults. LASA participants spent on average 65% of total wear time sedentary. This percentage was highest in those 80^+^ years (74.1%). LASA participants spent 25% performing light-low intensity activities, 8% light-high intensity activities, and 2% MVPA. Sedentary time and all physical activity intensity categories differed according to age, sex, education level and BMI categories. Time spent sedentary was gradually higher with older age, while light, moderate and vigorous physical activity were lower with older age. Higher age and higher BMI were related to more time sedentary, while female sex and lower education were related lower sedentary time. The joint associations of high sedentary time and low physical activity were associated with higher age, higher BMI, and slower walking speed compared to the combination of low sedentary time and high MVPA.

More time spent sedentary with higher age groups is in line with other studies that objectively measured various physical activity intensities in older adults all measured by hip-accelerometry [19, 21–23] or wrist-accelerometry [20]. In our study, women spent less time sedentary and more time in both light-low and light-high physical activity and less time in MVPA, which is also in line with most studies among older adults [5, 19, 21, 24]. An explanation for the higher light activity intensities in women might be traditional gender roles, in which women are more involved in household activities than men. In contrast, another Dutch cohort study found opposite results with higher MVPA in older women as assessed with a wrist wrist-worn accelerometer, [20] which is known to overestimate activities performed with the upper extremities [25].

Further, we observed clear differences between education levels. Sedentary time and light-high intensity physical activity was lower among lower educated participants, whereas the higher educated spent more time sedentary and more time in MVPA. A possible explanation might be that higher educated adults have a more sedentary lifestyle due to (previous) office work combined with more sport activities (the so called weekend-warriors) [26]. The higher educated participants had more often a paid job compared to the lower educated participants (31.9 vs 13.9%). Future research is needed to understand the observed difference in physical activity intensities among education categories.

Less time on all physical activity intensities were measured with higher BMI categories, particularly among overweight and obese participants compared to their normal weight counterparts, in agreement with existing data [19–21, 24].

Sedentary time was strongly, inversely correlated with the intensity categories light-low and light-high, while sedentary time was only modestly correlated with the moderate intensity category. This means that light activities are likely the inverse of sedentary time. Small changes towards light activity might be a good way to reduce sedentary time in older adults, without the burden of higher MVPA, which is not feasible for some older adults.

Important correlates of sedentary time were older age, female sex, lower education level, winter season, current smoking, higher BMI, high urbanization, faster walk test time, functional limitations and self-rated health. Importantly, living situation and the number of chronic diseases were not correlated with sedentary time and physical activity. Specifically, older age and higher BMI were the main correlates, which suggest a need to specifically develop interventions targeted at these individuals. The correlates of light-high to vigorous physical activity were mostly in the opposite direction compared to sedentary time.

Combined categories of sedentary and physical activity indicated clear differences between men and women. Men were more often high sedentary and high physically active, while women were more often low sedentary and low physically active, which could also be partly explained by the earlier mentioned traditional gender roles. Those with the most unfavorable physical activity pattern (high sedentary and low physically active) were older and had the highest BMI. Nevertheless, the low sedentary and low physical active group surprisingly had the lowest 6 m walk test.

A substantial group had worn the accelerometer for less than 10 h/day (600 min). This could be due to forgetting to put on the accelerometer, and due to hospital visits, swimming, or reported belly discomfort as reported in the diaries. In sensitivity analyses, we excluded these participants, which resulted in similar results. Therefore, we think that it is appropriate to include participants with less than 10 h of wear time per day since this group of older adults might have different daily routines including sleep and activity patterns.

### Strengths and limitations

An important strength of this study is the use of hip-accelerometry in a large population-based sample to objectively characterize minute-by-minute sedentary time and physical activity, and allowing to study different intensity levels (sedentary, light-low, light-high, moderate, and vigorous). This provides detailed data that cannot be gathered by traditional self-reported methods, particularly at low levels of physical activity. Also, accelerometry does not have recall-bias and social desirability bias and does not depend on literacy, which are the major limitations of self-report methods. Another strength is that accelerometer information was collected for 7 days for the majority (90%) of participants, which therefore truly reflects habitual behavior of older adults.

Some limitations have to be acknowledged as well. Accelerometers do not capture all types of physical activity, particular activities with a strong upper body component such as raking leaves or activities without a strong vertical component such as riding a bike (a common activity in the Netherlands) [25]. It is possibly that the intensity of bicycling was underestimated by the accelerometer, but it is not possible to determine if this resulted in underestimation of MVPA, and/or under or overestimation of light PA, and it is not feasible to correct objective physical activity data with self-reported physical activity data. Still the majority of participants (70%) reported bicycling activities in the past two weeks and therefore we included self-reported biking as a possible correlate in our models. Furthermore, water activities such as swimming during their data collection period were not registered as the accelerometer was removed, this led to some underestimation of physical activity with 9% of participants reporting swimming in the previous two weeks. Another limitation is possible selection bias (over- or under-recruitment of certain subgroups) for the cohort study as a whole, as well as for this particular sub-study. So the generalizability of the prevalence results to the general older population might be somewhat limited, while the reported associations are less prone to selection bias. There is also a risk for possible reactivity effects. Although, we cannot rule out that participants modified their activity behavior due to participation in the study, no studies to date have reported such an effects using 7-day accelerometer assessments.

In addition, the cut-off values for the higher intensity categories were developed for a general adult population, not for older adults [5, 27]. Particularly, the cut-point for MVPA may not apply to older populations and the optimal cut-off points may vary for different age-groups, due to dissimilar activity patterns, mechanical efficiency and the contrasting nature of movements at different life stages [28]. It is not unlikely that the used accelerometer cut-points misclassified moderate intensity physical activity as light intensity physical activity. For this reason, we divided the light intensity physical activity into light-low and light-high to better study the activity patterns of older adults. As MVPA levels are very low in older adults, the balance between sedentary time and light intensity activity is increasingly important when it comes to health and daily functioning. Which is another important reason why we looked at light intensity activity in more detail by splitting it into two groups. This increased focus on sedentary time and light intensity physical activity, next to MVPA, might be important to develop more effective guidelines, especially for older adults [15].

This study investigated both correlates of various physical activity intensities and joint combinations of different sedentary time and physical activity profiles across strata of sex, age, education and BMI groups. Sedentary time was strongly related with the intensity categories light-low and light-high, while sedentary time was only modestly related with moderate intensity physical activity meaning that these are activity patterns that are not strongly correlated or the inverse of each other. The high correlation between sedentary time and the two light intensity categories suggests that these are more likely to replace each other in daily routines. Changes in light intensity activities during daily routines as opposed to increase sports or other moderate to vigorous activities could lead to more substantial reductions in sedentary time and might be easier to implement. Nonetheless, the health benefits of moderate to vigorous intensity physical activity should not be overlooked and improving physical activity across the intensity spectrum is recommended.

## Conclusion

In our study, conducted in a population-based sample of Dutch older adults, the majority of wear time was spent sedentary 65% followed by light (33%), and MVPA (2%). Higher age and higher BMI were related to more time spent sedentary, while female sex and lower education were related to less time spent sedentary. Important correlates of sedentary time were older age, female sex, lower education level, winter season, current smoking, higher BMI, high urbanization, faster gait speed, more functional limitations and better self-rated health. The correlates of light-high to vigorous physical activity were mostly in the opposite direction compared to sedentary time.

The combination of high sedentary time (≥65.4% of waking time) and low physical (< 9.1% of waking time) was significantly associated with higher age, higher BMI, and slower walking speed compared to the combination of low sedentary time and high MVPA. Sedentary time was inversely correlated with light-intensity physical activity, but less so with MVPA. This suggests that increasing light activity might be an effective and feasible strategy in older persons to reduce sedentary time. Future studies should assess whether low- sedentary and high-light physical activity are associated with improved long-term health outcomes (also independent of MVPA).

## Methods

### Study population

We used data from the Longitudinal Aging Study Amsterdam (LASA), which is an ongoing population-based study in the Netherlands that started in 1992 to determine predictors and consequences of aging. A description of the cohort sampling and data collection procedures has been described elsewhere [29]. Briefly, 11 municipality registers from three geographical areas in the Netherlands were used to recruit men and women aged 55–85 years.

Since the start of LASA, two additional cohorts of participants aged 55–65 years were recruited using the same sampling frames exactly 10 (2002–2003) and 20 years (2012–2013) after the initial sample. For the current study, we used data from the 2015–2016 examination with participants of all three cohorts. At each examination, two interviews were conducted: a main interview and a medical interview with clinical measurements about 4–6 weeks apart.

**Fig. 2:**
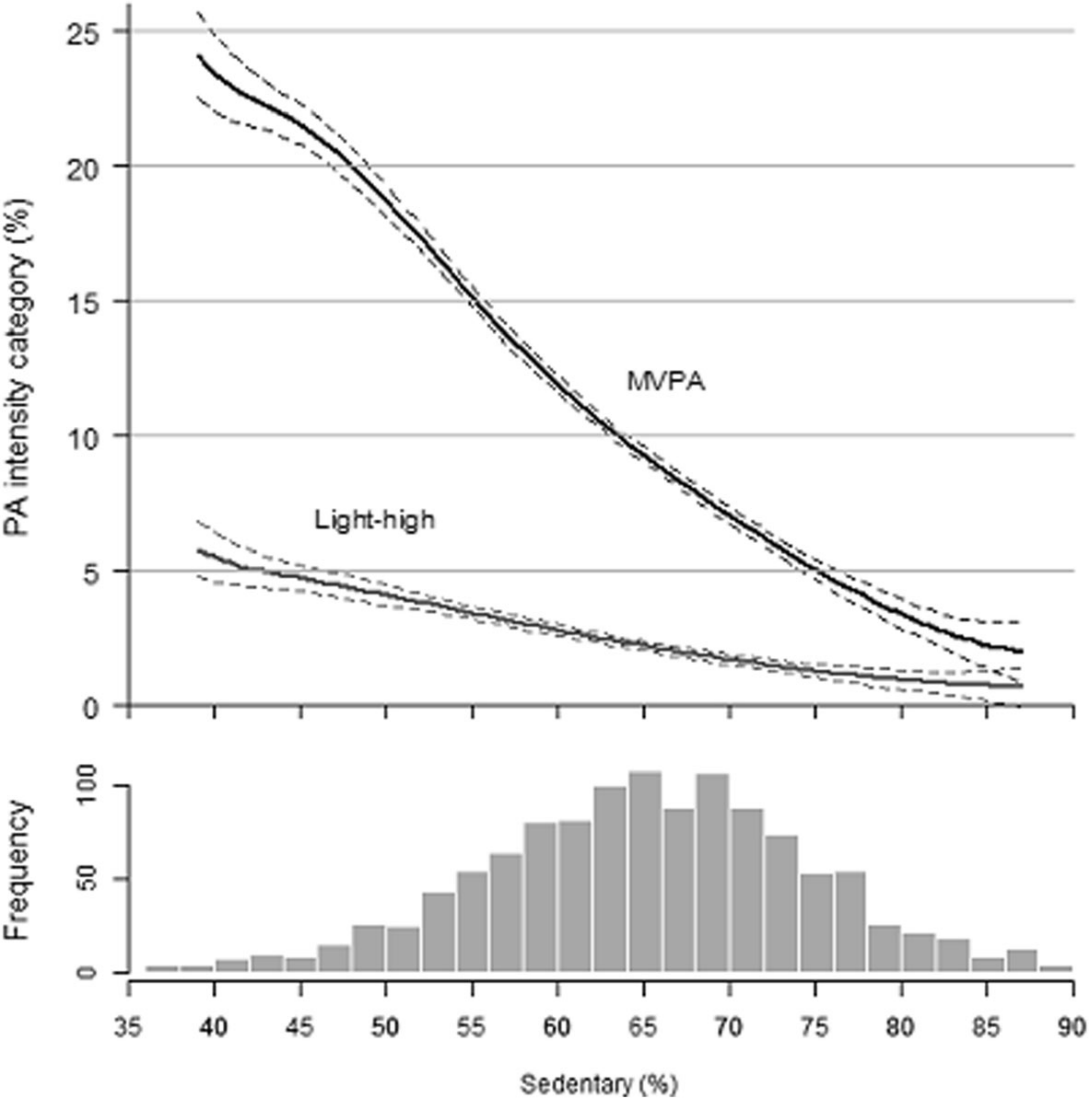
Continuous relationship between sedentary time with light-high intensity activity and MVPA expressed as percentage of wear time in 1201 LASA participants, adjusted for: age, sex, BMI, and education. Black lines: ≥760 cpm; gray lines: ≥2020 cpm. The bottom part of the figure shows the histogram depicting the sample distribution of sedentary time expressed as (%) of wear-time. The upper part of the graph represents the inverse relationship between sedentary time with the intensity categories light-high and MVPA. Black lines: ≥760 cpm; gray lines: ≥2020 cpm.”

All 1770 LASA participants who participated in the main interview were invited for the accelerometry ancillary study (See flow diagram Fig. 2). A total of 1412 participants indicated to be willing to participate and were sent an accelerometer for a 7-day period of whom 1218 wore and sent back the accelerometer (response 86%). Reasons of non-participations were: acute health problems (*n* = 151), lost accelerometer (*n* = 24), measurement error/broken accelerometer (*n* = 4), too frail (n = 2), too busy (n = 2), or unknown (*n* = 11).

For the present analysis, we excluded (*n* = 17) participants who wore the accelerometer < 4 days. Of all participants, 4 occasionally used a wheelchair, 10 used a walking stick and 7 used a walker, but all were able to walk. Altogether 1201 LASA participants had valid accelerometry data and formed the analytic sample. The LASA study is conducted in line with the Declaration of Helsinki, and was approved by the medical ethics committee of the VU University medical center.

### Objective measurement of physical activity

An accelerometer is a small device that records the acceleration of the body. The Actigraph tri-axial accelerometer (Model GT3X+; ActiGraph, Pensacola, USA) was used to objectively measure participants’ physical activity intensities. The accelerometer together with an instruction brochure with pictures how to properly wear the accelerometer was sent to the participants by regular mail. The accelerometer was worn around the waist with an elastic belt and placed above the right iliac crest for comparability with most other studies that assessed objective physical activity. Participants were instructed to wear the accelerometer for a consecutive 7-day period during waking hours with the exception of water based activities such as bathing, showering and swimming.

Participants completed a daily log diary to record the time after waking up that the accelerometer was put on and the time the accelerometer was taken off just before going to bed, as well as the times and the reason when the accelerometer was taken off during the day. The log diaries were used to indicate participants with non-wear time due to other activities. Participants were instructed to wear the accelerometer only during waking hours, however some participants forgot to or experienced difficulties taking-off the accelerometer (*n* = 19). For these participants, wear time and sedentary time was adjusted using the sleep information from the log diary. Adjusted wear time was calculated as: 1440 (total minutes per day) – reported sleep time assuming that a participant wears the accelerometer during all wear hours. The adjusted sedentary time for this small group was calculated as: adjusted wear time – low light – light high – moderate – vigorous physical activity.

The accelerometer data were processed with ActiLife 6.13.3 (Actigraph, Pensacola, USA). Accelerometer data was collected using 1 s epochs and aggregated to 60 s epochs for data reduction. Data periods with consecutive zero counts for ≥60 min, with allowance for 1–2 min of counts between 0 and 100, were considered as non-wear time periods. A minimum of four valid days was needed for a participant to be included in the analyses. Physical activity intensity categories were defined according to the following existing accelerometer cut-points for activity counts per minute (cpm) [5, 27, 30].

- Sedentary: < 100 cpm
- Light: ≥ 100 and < 2020 cpm
  - Light-low: ≥ 100 and < 760 cpm
  - Light-high: ≥ 760 and < 2020 cpm
- Moderate: ≥ 2020 and < 5999 cpm
- Vigorous ≥5999 cpm

The intensity categories moderate and vigorous physical activity were also summed (MVPA). We added the light-low and light-high categories for a better insight in the distribution of the different intensity levels of older participants. In this population, light-high activities might have been of a moderate intensity for some participants.

### Other variables

LASA interviewers obtained comprehensive data on participants’ demographics, anthropometrics and co-morbid conditions during the main interview. Body height was measured to the nearest 0.1 cm using a stadiometer. Body weight was measured without clothes and shoes to the nearest 0.1 kg using a calibrated bathroom scale (Seca, model 100, Lameris, Utrecht, the Netherlands). When necessary, corrections were made to adjust measured body weight for clothing (− 2 kg) or corset (− 1 kg). Body mass index (BMI) was calculated by dividing body weight by height squared (kg/m^2^). We defined BMI categories as: underweight < 70 years < 18.5 kg/m^2^, ≥ 70 years < 20 kg/m^2^, normal: ≥20–25 kg/m^2^, overweight ≥25–30 kg/m^2^, and obese ≥30 kg/m^2^ [31].

The interviewer assessed participants’ education level, smoking status, and living situation. Education was reported on a 9-category scale. We distinguished education into 3 categories: low (elementary school or less), medium (lower vocational or general intermediate education) and high (intermediate vocational education, general secondary school, higher vocational education, college or university). Smoking status was categorized as never, former and current smoker. Living situation was defined as living alone or together with a spouse/partner/family member. Self-rated health was assessed as a measure of overall health status with 4 response categories. We dichotomized this question to poor/fair and good/excellent. Season was calculated based on the first day the accelerometer was worn using meteorological seasons divided into autumn, winter, spring and summer. Level of urbanization was assessed based on the number of addresses per km^2^ (rural, < 1000 addresses/km^2^; intermediate, 1000–2500 addresses/km^2^; urban, ≥ 2500 addresses/km^2^).

The number of chronic diseases was based on self-report of the most frequent somatic chronic diseases in the Netherlands and included: chronic non-specific lung disease, cardiac disease, peripheral artery disease, stroke, type 2 diabetes, arthritis and malignancies. Self-reported functional limitations was measured with a questionnaire adapted from the Organization for Economic Cooperation and Development (OECD) questionnaire and validated by Central Bureau of Statistics Netherlands [29]. Participants were asked whether they have difficulty performing 4 common activities related to mobility: 1) walk up and down a 15-step staircase without resting, 2) sit down and get up from a chair, 3) walk 5 min outside without resting, 4) drive or use public transport. The total score ranged from no limitation to limitations for all functions (stairs/transport/chair/walk) score 0–4 and was categorized into 0 limitations and ≥ 1 limitation. Further, self-reported bicycling and swimming in the past two weeks was measured with the LASA physical activity questionnaire [32].

For the 6 m walk test, participants were asked to walk 3 m, turn 180°, and walk back 3 m as fast as possible while the interviewer recorded the time in seconds. A higher walk test time indicates poorer physical performance.

### Statistical analyses

Baseline characteristics are presented as mean and standard deviation for continuous variables or number and percentage for categorical variables. We summarized demographics, lifestyle, health measures, sedentary time and physical activity (total and different intensity categories) by sex and calculated Pearson correlation coefficients among various physical activity intensity categories.

We graphically displayed the continuous relationship for sedentary time and physical activity intensities as percentage of total wear time using cubic splines with 95% confidence intervals adjusting for age, sex, BMI and education level. We used analysis of covariance to estimate adjusted means and 95% confidence intervals to study characteristics of 7-day hip accelerometry by categories of age, sex, education and BMI using the F-test as P-for trend over the adjusted means.

Further, we applied hierarchical regression analysis to assess correlates of sedentary time and physical activity with a *P*-value of 0.10 as inclusion criterion using complete case analysis. The basic model included age, sex and education as a block of covariates and correlates were added one by one thereafter. In total three blocks were defined: block 1 age, sex, education and season; block 2: adds smoking, BMI, and urbanization categories; block 3 adds walking speed, functional limitations and self-rated health. The results are reported as unstandardized regression coefficients with 95% confidence intervals. In addition, the R^2^ was assessed to estimate the explained variance of sedentary time or a specific physical activity intensity.

Next, we estimated adjusted means across combined sedentary and physical activity categories based on the median using analysis of covariance.

### Sensitivity analyses

Additionally, we performed a sensitivity analysis to test the robustness of the associations. Participants who wore the accelerometer at night (*n* = 19), participants with a mean wear time < 600 min for ≥4 days (*n* = 136) and participants who reported a significant break in wear time based on self-report form the log diary (*n* = 144) were excluded.

Statistical analyses were performed with SPSS for Windows (version 22.0).

## Additional file

Additional file 1: Supplemental Tables. (DOCX 38.1 kb)
