# Supplementary material for: Daily sedentary time and physical activity as assessed by accelerometry and their correlates in older adults
Source: Eur Rev Aging Phys Act. 2019 Feb 18;16:3. doi: 10.1186/s11556-019-0210-9 (PMC6379946; doi:10.1186/s11556-019-0210-9)
Supplement: Supplementary file 1 — Supplemental Tables. (DOCX 38.1 kb) [file 11556_2019_210_MOESM1_ESM.docx]

**Supplemental tables**

**Table S1**: Hierarchical regression analysis of correlates of moderate and vigorous activity as percentage of total wear time in 1,201 LASA participants

|  | **Block 1** | | | **Block 2** | | | **Block 3** | | |
| --- | --- | --- | --- | --- | --- | --- | --- | --- | --- |
|  | β | SE | P-value | β | SE | P-value | β | SE | P-value |
| *Demographic* |  |  |  |  |  |  |  |  |  |
| Age ≤65 years (ref) | 0.0 |  |  | 0.0 |  |  | 0.0 |  |  |
| Age 65-70 years | -0.3 | 0.2 | 0.097 | -0.4 | 0.2 | 0.040 | -0.3 | 0.2 | 0.099 |
| Age 70-75 years | -1.0 | 0.2 | <0.001 | -1.1 | 0.2 | <0.001 | -1.0 | 0.2 | <0.001 |
| Age 75-80 years | -1.3 | 0.2 | <0.001 | -1.4 | 0.2 | <0.001 | -1.3 | 0.2 | <0.001 |
| Age ≥80 years | -2.3 | 0.2 | <0.001 | -2.5 | 0.2 | <0.001 | -2.0 | 0.2 | <0.001 |
| Men (ref) | 0.0 |  |  | 0.0 |  |  | 0.0 |  |  |
| Women | -0.6 | 0.1 | <0.001 | -0.8 | 0.1 | <0.001 | -0.7 | 0.1 | <0.001 |
| Education low | 0.1 | 0.2 | 0.418 | 0.2 | 0.2 | 0.294 | 0.3 | 0.2 | 0.087 |
| Education medium | -0.8 | 0.2 | <0.001 | -0.6 | 0.2 | <0.001 | -0.5 | 0.2 | 0.001 |
| Education high (ref) | 0.0 |  |  | 0.0 |  |  | 0.0 |  |  |
| Spring (ref) | 0.0 |  |  | 0.0 |  |  | 0.0 |  |  |
| Summer | -0.1 | 0.2 | 0.472 | -0.2 | 0.2 | 0.270 | 0.2 | 0.2 | 0.391 |
| Autumn | -0.2 | 0.2 | 0.260 | -0.2 | 0.2 | 0.219 | -0.2 | 0.2 | 0.237 |
| Winter | -0.3 | 0.2 | 0.086 | -0.3 | 0.2 | 0.068 | -0.3 | 0.2 | 0.093 |
| *Lifestyle* |  |  |  |  |  |  |  |  |  |
| Never smoker (ref) |  |  |  | 0.0 |  |  | 0.0 |  |  |
| Former smoker |  |  |  | 0.1 | 0.1 | 0.424 | 0.1 | 0.1 | 0.461 |
| Current smoker |  |  |  | -0.6 | 0.2 | 0.009 | -0.5 | 0.2 | 0.033 |
| BMI category underweight |  |  |  | 0.4 | 0.2 | 0.052 | 0.3 | 0.2 | 0.107 |
| BMI category normal weight (ref) |  |  |  | 0.0 |  |  | 0.0 |  |  |
| BMI category overweight |  |  |  | -0.8 | 0.2 | <0.001 | -0.7 | 0.2 | <0.001 |
| BMI category obese |  |  |  | -1.3 | 0.3 | <0.001 | -1.0 | 0.3 | 0.002 |
| *Health measures* |  |  |  |  |  |  |  |  |  |
| Duration 6 meter walk test (s) |  |  |  |  |  |  | -0.1 | 0.1 | 0.007 |
| No functional limitation (ref) |  |  |  |  |  |  | 0.0 |  |  |
| ≥ l functional limitation |  |  |  |  |  |  | -0.4 | 0.2 | 0.014 |
| Self-rated health poor/fair |  |  |  |  |  |  | -0.4 | 0.2 | 0.013 |
| Self-rated health good/excellent (ref) |  |  |  |  |  |  | 0.0 |  |  |
|  |  |  |  |  |  |  |  |  |  |
| Explained variance (R^2^) | 0.156 |  | <0.001 | 0.195 |  | <0.001 | 0.217 |  | <0.001 |

Unstandardized regression coefficients and standard errors (SE)

Physical activity: ≥2020 cpm (combined moderate and vigorous)

Ref: Reference. Lower values imply less time spent in physical activity

Consecutive blocks of correlates:

block 1: non-modifiable and season

block 2 lifestyle

block 3: health measures

Number of chronic diseases and urbanization were not significant correlates

Consecutive blocks of correlates; block 1: non-modifiable and season, block 2 lifestyle block 3: health measures

**Table S2**: Hierarchical regression analysis of correlates of light-high to vigorous intensity physical activity* in 1,201 LASA participants

|  | **Block 1** | | | **Block 2** | | | **Block 3** | | |
| --- | --- | --- | --- | --- | --- | --- | --- | --- | --- |
|  | β | SE | P-value | β | SE | P-value | β | SE | P-value |
| *Demographic factors* |  |  |  |  |  |  |  |  |  |
| Age ≤65 years (ref) | 0.0 |  |  | 0.0 |  |  | 0.0 |  |  |
| Age 65-70 years | -1.3 | 0.4 | 0.003 | -1.4 | 0.4 | 0.001 | -1.1 | 0.4 | 0.007 |
| Age 70-75 years | -2.5 | 0.5 | <0.001 | -2.7 | 0.5 | <0.001 | -2.3 | 0.5 | <0.001 |
| Age 75-80 years | -3.2 | 0.5 | <0.001 | -3.5 | 0.5 | <0.001 | -2.9 | 0.5 | <0.001 |
| Age ≥80 years | -7.6 | 0.5 | <0.001 | -8.0 | 0.6 | <0.001 | -6.3 | 0.6 | <0.001 |
| Men (ref) | 0.0 |  |  | 0.0 |  |  | 0.0 |  |  |
| Women | -0.5 | 0.3 | 0.102 | -0.7 | 0.3 | 0.043 | -0.3 | 0.3 | <0.001 |
| Education low | 1.1 | 0.4 | 0.006 | 1.1 | 0.4 | 0.004 | 1.5 | 0.4 | <0.001 |
| Education medium | -0.6 | 0.4 | 0.124 | -0.4 | 0.4 | 0.377 | -0.1 | 0.4 | 0.823 |
| Education high (ref) | 0.0 |  |  | 0.0 |  |  | 0.0 |  |  |
| Spring (ref) | 0.0 |  |  | 0.0 |  |  | 0.0 |  |  |
| Summer | -0.1 | 0.5 | 0.914 | -0.1 | 0.5 | 0.780 | 0.1 | 0.5 | 0.937 |
| Autumn | -0.9 | 0.5 | 0.055 | -1.2 | 0.5 | 0.013 | -1.1 | 0.5 | 0.017 |
| Winter | -1.5 | 0.4 | 0.001 | -1.5 | 0.4 | 0.001 | -1.4 | 0.4 | 0.001 |
| *Lifestyle factors* |  |  |  |  |  |  |  |  |  |
| Never smoker (ref) |  |  |  | 0.0 |  |  | 0.0 |  |  |
| Former smoker |  |  |  | 0.4 | 0.4 | 0.295 | 0.3 | 0.4 | 0.339 |
| Current smoker |  |  |  | -1.6 | 0.6 | 0.007 | -1.2 | 0.6 | 0.039 |
| BMI category underweight |  |  |  | -0.1 | 0.5 | 0.885 | -0.3 | 0.5 | 0.522 |
| BMI category normal weight (ref) |  |  |  | 0.0 |  |  | 0.0 |  |  |
| BMI category overweight |  |  |  | -4.7 | 1.5 | 0.003 | -3.9 | 1.6 | 0.012 |
| BMI category obese |  |  |  | -4.0 | 1.0 | <0.001 | -2.8 | 1.0 | 0.004 |
| Urbanization low (ref) |  |  |  | 0.0 |  |  | 0.0 |  |  |
| Urbanization intermediate |  |  |  | -0.1 | 0.3 | 0.827 | -0.1 | 0.3 | 0.678 |
| Urban high |  |  |  | 3.2 | 1.5 | 0.037 | 2.8 | 1.5 | 0.058 |
| *Health and function factors* |  |  |  |  |  |  |  |  |  |
| Duration 6 meter walk test (s) |  |  |  |  |  |  | -0.2 | 0.1 | <0.001 |
| No functional limitation (ref) |  |  |  |  |  |  | 0.0 |  |  |
| ≥ l functional limitation |  |  |  |  |  |  | -1.1 | 0.4 | 0.006 |
| Bicycling (yes) |  |  |  |  |  |  | 0.4 | 0.1 | <0.001 |
| Self-rated health poor/fair |  |  |  |  |  |  | -1.5 | 0.4 | <0.001 |
| Self-rated health good/excellent (ref) |  |  |  |  |  |  | 0.0 |  |  |
|  |  |  |  |  |  |  |  |  |  |
| Explained variance (R^2^) | 0.176 |  | <0.001 | 0.203 |  | <0.001 | 0.258 |  | <0.001 |

Unstandardized regression coefficients and standard errors (SE)

* ≥760 cpm which includes light-high, moderate and vigorous intensity activity, expressed as percentage of total wear time

Ref: Reference. Lower values imply less time spent in physical activity

Consecutive blocks of correlates: block 1non-modifiable factors and season; block 2 lifestyle factors; block 3 health and function factors. Living situation and number of chronic diseases were not significant correlates.

**Table S3**: Adjusted means across four combined sedentary time and (moderate to vigorous intensity*) physical activity profiles among 1,201 LASA participants

|  | **High sedentary**  **Low MVPA**  **N=403** | **High sedentary**  **High MVPA**  **N=197** | **Low sedentary**  **Low MVPA**  **N=195** | **Low sedentary**  **High MVPA**  **N=406** | **P-value** |
| --- | --- | --- | --- | --- | --- |
| Men  Women | 187 (46%)  216 (54%) | 136 (69%)  61 (31%) | 58 (30%)  137 (70%) | 205 (50%)  201 (50%) |  |
| **ANCOVA** |  |  |  |  |  |
| Age (years) | 73.2 (72.4-74.0) | 68.7 (67.6-69.7) | 72.1 (71.0-73.2) | 68.3 (67.6-69.0) | <0.001 |
| BMI (kg/m^2^) | 23.7 (23.3-24.1) | 22.2 (21.7-22.7) | 23.7 (23.1-24.3) | 22.1 (21.7-22.5) | <0.001 |
| 6 meter walk test (sec) | 7.7 (7.3-8.0) | 6.3 (5.8-6.7) | 7.5 (7.0-8.0) | 7.1 (6.8-7.4) | <0.001 |
| Light-low (%) | 22.1 (21.6-22.5) | 20.8 (20.2-21.3) | 30.6 (30.0-31.2) | 26.9 (26.5-27.3) | <0.001 |

MVPA: moderate and vigorous physical activity intensity category

*Categorization based on median: sedentary <100 (cpm) <65.4/≥65.4%, moderate to vigorous ≥2020 (cpm) <1.7/≥1.7% out of total wear time

ANCOVA: analysis of covariance with 95% confidence intervals. Significant P<0.05 when no overlap occurs in 95% confidence interval between categories.

Adjusted for sex, education categories, age, BMI and 6 meter walk test.

**Table S4:** Characteristics of LASA 7 day hip-accelerometer study of 904 LASA participants, excluding participants with night wear, with a mean wear time <600 minutes for ≥ 4 days and participants who reported a significant break in wear time were excluded.

|  | **Total group** | **Men** | **Women** |
| --- | --- | --- | --- |
| **Demographics** |  |  |  |
| N | 904 | 451 | 453 |
| Mean age | 71.2±8.1 | 70.7±7.9 | 71.8±8.4 |
| Age categories  <65 years  65-70 years  70-75 years  75-80 years  ≥80 years | 224 (25%)  244 (27%)  163 (18%)  129 (14%)  144 (16%) | 113 (25%)  136 (30%)  81 (18%)  60 (13%)  61 (14%) | 111 (25%)  108 (24%)  82 (18%)  69 (15%)  83 (18%) |
| Education  Low  Middle  High | 301 (33%)  345(38%)  258 (29%) | 134 (30%)  153 (34%)  164 (36%) | 167 (37%)  192 (42%)  94 (21%) |
| Urbanization grade  Rural  Intermediate urban  Urban | 400 (44%)  339(38%)  164 (18%) | 192 (43%)  182 (40%)  77 (17%) | 208 (46%)  157 (35%)  87 (19%) |
| Living situation  Alone  Together | 262 (29%)  642 (71%) | 83 (18%)  368 (82%) | 179 (40%)  274 (60%) |
| Season  Summer  Fall  Winter  Spring | 183 (20%)  210 (23%)  261 (29%)  250 (28%) | 84 (19%)  115 (25%)  132 (29%)  120 (27%) | 99 (22%)  95 (21%  129 (29%)  130 (29%) |
| **Lifestyle** |  |  |  |
| Smoking  Current  Former  Never | 93 (10%)  557 (63%)  239 (27%) | 49 (11%)  300 (68%)  93 (21%) | 44 (10%)  257 (58%)  146 (32%) |
| BMI categories*  Underweight  Normal  Overweight  Obese | 156 (17%)  511 (58%)  186 (21%)  33 (4%) | 33 (7%)  269 (61%)  118 (27%)  20 (5%) | 123 (28%)  242 (54%)  68 (15%)  13 (3%) |
| **Health measures** |  |  |  |
| Number of chronic diseases  0  1  ≥2 | 198 (22%)  276 (30%)  430 (48%) | 113 (25%)  147 (33%)  191 (42%) | 85 (19%)  129 (29%)  239 (53%) |
| Self-rated health  Excellent/good  Poor/fair | 670 (74%)  233 (26%) | 353 (78%)  98 (22%) | 317 (70%)  135 (30%) |
| ≥ 1 functional limitation | 309 (34%) | 134 (30%) | 175 (39%) |
| 6 meter walk test (sec) | 7.3±3.2 | 7.00±2.8 | 7.6±3.5 |
| Bicycling | 625 (69%) | 324 (72%) | 301 (66%) |
| Swimming | 3 (0.3%) | 1 (0.2%) | 2 (0.4%) |
| **Accelerometry** |  |  |  |
| Wear time (min/d) | 854±77 | 858±78 | 850±77 |
| Sedentary time (min/d) | 554±85 (65%) | 566±81 (66.4%) | 543±86 (64.1%) |
| Light-low time (min/d) | 213±58 (25%) | 201±53 (23.3%) | 225±61(26.3%) |
| Light-high time (min/d) | 65±43 (7.5%) | 66±45 (7.5%) | 65±41 (7.5%) |
| Moderate time (min/d) | 14 (5-28) (2.3%) | 17 (7-32) (2.6%) | 11 (3-23) (2.0%) |
| Vigorous time (min/d) | 0.0 (0-0) (0.1%) | 0.0 (0-0) (0.1%) | 0.0 (0-0) (0.0%) |

Mean±standard deviation median and interquartile range or number and percentage;

For accelerometertry data: mean time±SD plus % out of total wear time.

Self-reported functional limitations: scale 0-100, BMI categories: <70 years BMI <18.5 kg/m^2^ underweight, ≥ 70 years BMI <20 kg/m^2^ underweight

**Table S5:** Characteristics of 7-day hip-accelerometry by age, sex, education and body mass index groups in 904 LASA participants

|  | **Wear time (min)** | **Sedentary (%)** | **Light-low (%)** | **Light-high (%)** | **MVPA (%)** |
| --- | --- | --- | --- | --- | --- |
| **Age categories**  <65 years  65-70 years  70-75 years  75-80 years  ≥80 years  *P*-trend | 880 (869-889)  856 (847-865)  859 (847-870)  844 (831-857)  813 (800-826)  <0.001 | 63.4 (62.3-64.5)  64.1 (63.0-65.1)  64.4 (63.1-65.6)  65.8 (64.3-67.2)  71.6 (70.2-73.0) <0.001 | 25.8 (25.1-26.6)  25.6 (24.9-26.3)  25.6 (24.7-26.4)  24.4 (23.4-25.4)  21.1 (20.1-22.1)  <0.001 | 8.4 (7.9-8.9)  8.0 (7.4-8.5)  7.7 (7.1-8.3)  7.5 (6.8-8.2 )  4.9 (4.2-5.6)  <0.001 | 2.8 (2.6-3.1)  2.7 (2.4-2.9)  2.0 (1.7-2.3)  2.0 (1.6-2.3)  1.7 (1.4-2.1)  <0.001 |
| **Sex**  Men  Women  *P*-trend | 855 (848-862)  852 (846-860)  0.696 | 67.2 (66.4-68.0)  63.6 (62.8-64.4)  <0.001 | 23.4 (22.8-23.9)  26.2 (25.6-26.7)  <0.001 | 7.1 (6.7-7.5)  7.9 (7.5-8.2)  0.007 | 2.8 (2.6-3.0)  1.9 (1.7-2.1)  <0.001 |
| **Education**  Low  Middle  High  *P*-trend | 854 (846-863)  848 (840-856)  860 (851-869)  0.175 | 64.0 (63.0-64.9)  65.4 (64.6-66.3)  67.0 (66.0-68.1)  <0.001 | 25.5 (24.9-26.2)  24.8 (24.2-25.4)  23.8 (23.2-24.5)  0.003 | 8.2 (7.7-8.6)  7.4 (7.0-7.8)  6.8 (6.3-7.3)  <0.001 | 2.1 (1.9-2.3)  2.1 (1.9-2.4)  2.9 (2.7-3.2)  <0.001 |
| **BMI categories***  Underweight  Normal  Overweight  Obese  *P*-trend | 858 (846-870)  855 (849-862)  845 (835-857)  850 (825-876)  0.432 | 65.0 (63.7-66.4)  64.9 (64.2-65.6)  66.5 (65.3-67.7)  68.3 (65.5-71.2)  0.030 | 25.8 (24.8-26.7)  24.7 (24.7-25.7)  23.4 (22.6-24.2)  21.7 (19.8-23.6)  <0.001 | 6.8 (6.2-7.5)  7.6 (7.2-7.9)  7.5 (7.1-8.3)  7.6 (6.3-9.0)  0.182 | 2.6 (2.3-3.0)  2.5 (2.3-2.6)  1.9 (1.6-2.2)  1.6 (0.9-2.3) 0.001 |

Analyzed with analysis of covariance with 95% confidence intervals. Significant P<0.05when no overlap occurs in 95% confidence interval between categories. Adjusted for all other variables listed plus MVPA. For MVPA all variables listed plus sedentary time.

*BMI categories: <70 years BMI <18.5 kg/m^2^ underweight, ≥ 70 years BMI <20 kg/m^2^ underweight

**Table S6:** Hierarchical regression analysis of correlates of sedentary intensity as percentage of total wear time in 904 LASA participants

|  | **Block 1** | | | **Block 2** | | | **Block 3** | | |
| --- | --- | --- | --- | --- | --- | --- | --- | --- | --- |
|  | β | SE | P-value | β | SE | P-value | β | SE | P-value |
| *Demographic* |  |  |  |  |  |  |  |  |  |
| Age ≤65 years (ref) | 0.0 |  |  | 0.0 |  |  | 0.0 |  |  |
| Age 65-70 years | 1.2 | 0.9 | 0.172 | 1.1 | 0.8 | 0.192 | 0.6 | 0.8 | 0.453 |
| Age 70-75 years | 2.3 | 1.0 | 0.020 | 2.3 | 1.0 | 0.015 | 1.6 | 0.9 | 0.092 |
| Age 75-80 years | 4.2 | 1.0 | <0.001 | 4.4 | 1.0 | <0.001 | 3.4 | 1.0 | <0.001 |
| Age ≥80 years | 11.7 | 1.0 | <0.001 | 12.2 | 1.0 | <0.001 | 8.8 | 1.1 | <0.001 |
| Men (ref) | 0.0 |  |  | 0.0 |  |  | 0.0 |  |  |
| Women | -2.6 | 0.6 | <0.001 | -2.0 | 0.6 | 0.002 | -2.6 | 0.6 | <0.001 |
| Education low | -1.5 | 0.7 | 0.048 | -1.6 | 0.7 | 0.032 | -2.6 | 0.7 | <0.001 |
| Education medium | -0.3 | 0.8 | 0.700 | -0.9 | 0.8 | 0.235 | -1.3 | 0.7 | 0.080 |
| Education high (ref) | 0.0 |  |  | 0.0 |  |  | 0.0 |  |  |
| Spring (ref) | 0.0 |  |  | 0.0 |  |  | 0.0 |  |  |
| Summer | -0.8 | 0.9 | 0.351 | -0.8 | 0.9 | 0.376 | -1.2 | 0.9 | 0.166 |
| Autumn | 0.9 | 0.9 | 0.297 | 1.5 | 0.9 | 0.088 | 1.4 | 0.8 | 0.109 |
| Winter | 2.3 | 0.8 | 0.005 | 2.5 | 0.8 | 0.002 | 2.2 | 0.8 | 0.006 |
| *Lifestyle* |  |  |  |  |  |  |  |  |  |
| Never smoker (ref) |  |  |  | 0.0 |  |  | 0.0 |  |  |
| Former smoker |  |  |  | -0.2 | 0.7 | 0.787 | -0.2 | 0.7 | 0.744 |
| Current smoker |  |  |  | 3.4 | 1.1 | 0.003 | 2.2 | 1.1 | 0.044 |
| BMI category underweight |  |  |  | -0.4 | 0.9 | 0.672 | -0.1 | 0.8 | 0.991 |
| BMI category normal weight (ref) |  |  |  | 0.0 |  |  | 0.0 |  |  |
| BMI category overweight |  |  |  | 11.4 | 3.2 | <0.001 | 9.8 | 3.1 | 0.002 |
| BMI category obese |  |  |  | 9.1 | 1.9 | <0.001 | 7.7 | 1.9 | <0.001 |
| Urbanization low (ref) |  |  |  | 0.0 |  |  | 0.0 |  |  |
| Urbanization intermediate |  |  |  | -0.4 | 0.6 | 0.529 | -0.4 | 0.6 | 0.527 |
| Urban high |  |  |  | -8.1 | 3.2 | 0.010 | -7.4 | 3.0 | 0.016 |
| *Health measures* |  |  |  |  |  |  |  |  |  |
| Duration 6 meter walk test (s) |  |  |  |  |  |  | 0.6 | 0.1 | <0.001 |
| No functional limitation (ref) |  |  |  |  |  |  | 0.0 |  |  |
| ≥ l functional limitation |  |  |  |  |  |  | 1.4 | 0.7 | 0.063 |
| Bicycling (yes) |  |  |  |  |  |  | -1.0 | 0.2 | <0.001 |
| Self-rated health poor/fair |  |  |  |  |  |  | 2.6 | 0.8 | <0.001 |
| Self-rated health good/excellent (ref) |  |  |  |  |  |  | 0.0 |  |  |
|  |  |  |  |  |  |  |  |  |  |
| Explained variance (R^2^) | 0.179 |  | <0.001 | 0.220 |  | <0.001 | 0.286 |  | <0.001 |

Ref: Reference

Unstandardized regression coefficients and standard errors (SE)

Consecutive blocks of correlates; block 1: non-modifiable and season, block 2 lifestyle block 3: health measures

**Table S7**: Hierarchical regression analysis of correlates of physical activity as percentage of total wear time in 904 LASA participants

|  | **Block 1** | | | **Block 2** | | | **Block 3** | | |
| --- | --- | --- | --- | --- | --- | --- | --- | --- | --- |
|  | β | SE | P-value | β | SE | P-value | β | SE | P-value |
| *Demographic* |  |  |  |  |  |  |  |  |  |
| Age ≤65 years (ref) | 0.0 |  |  | 0.0 |  |  | 0.0 |  |  |
| Age 65-70 years | -1.1 | 0.5 | 0.032 | -1.1 | 0.5 | 0.040 | -0.8 | 0.6 | <0.001 |
| Age 70-75 years | -2.6 | 0.6 | <0.001 | -2.5 | 0.5 | <0.001 | -2.1 | 0.6 | <0.001 |
| Age 75-80 years | -3.2 | 0.6 | <0.001 | -3.3 | 0.6 | <0.001 | -2.8 | 0.6 | <0.001 |
| Age ≥80 years | -7.6 | 0.6 | <0.001 | -7.8 | 0.6 | <0.001 | -6.1 | 0.7 | <0.001 |
| Men (ref) | 0.0 |  |  | 0.0 |  |  | 0.0 |  |  |
| Women | -0.4 | 0.4 | 0.246 | -0.6 | 0.4 | 0.134 | -0.3 | 0.4 | 0.467 |
| Summer | 0.2 | 0.6 | 0.740 | 0.2 | 0.6 | 0.753 | 0.4 | 0.5 | 0.445 |
| Autumn | -0.9 | 0.5 | 0.109 | -1.2 | 0.5 | 0.026 | -1.1 | 0.5 | 0.034 |
| Winter | -1.5 | 0.5 | 0.003 | -1.6 | 0.5 | 0.001 | -1.4 | 0.5 | 0.003 |
| *Lifestyle* |  |  |  |  |  |  |  |  |  |
| Never smoker (ref) |  |  |  | 0.0 |  |  | 0.0 |  |  |
| Former smoker |  |  |  | 0.3 | 0.4 | 0.518 | 0.3 | 0.4 | 0.518 |
| Current smoker |  |  |  | -1.4 | 0.7 | 0.046 | -1.4 | 0.5 | 0.046 |
| BMI category underweight |  |  |  | -0.3 | 0.5 | 0.635 |  |  |  |
| BMI normal category (ref) |  |  |  | 0.0 |  |  | 0.0 |  |  |
| BMI category overweight |  |  |  | -5.4 | 2.0 | 0.006 | -5.4 | 2.0 | 0.006 |
| BMI category obese |  |  |  | 3.6 | 1.2 | <0.002 | -3.6 | 1.2 | 0.002 |
| Urbanization low (ref) |  |  |  | 0.0 |  |  | 0.0 |  |  |
| Urbanization intermediate |  |  |  | 0.1 | 0.4 | 0.905 | 0.1 | 0.4 | 0.900 |
| Urban high |  |  |  | 4.9 | 2.0 | 0.013 | 4.4 | 1.9 | 0.021 |
| *Health measures* |  |  |  |  |  |  |  |  |  |
| Duration 6 meter walk test (s) |  |  |  |  |  |  | -0.3 | 0.1 | <0.001 |
| No functional limitation (ref) |  |  |  |  |  |  | 0.0 |  |  |
| ≥ l functional limitation |  |  |  |  |  |  | -0.9 | 0.5 | 0.048 |
| Bicycling (yes) |  |  |  |  |  |  | 0.5 | 0.1 | <0.001 |
| Self-rated health poor/fair |  |  |  |  |  |  | -1.6 | 0.5 | <0.001 |
| Self-rated health good/excellent (ref) |  |  |  |  |  |  | 0.0 |  |  |
|  |  |  |  |  |  |  |  |  |  |
| Explained variance (R^2^) | 0.174 |  | <0.001 | 0.196 |  | <0.001 | 0.271 |  | <0.001 |

Physical activity: combined light-high, moderate and vigorous intensity category

Ref: Reference

Unstandardized regression coefficients and standard errors (SE)

Consecutive blocks of correlates; block 1: non-modifiable and season, block 2 lifestyle block 3: health measures

**Table S8**: Adjusted means across four combined sedentary time and (light-high to vigorous*) intensity physical activity profiles in 904 LASA participants

|  | **High sedentary**  **Low PA**  **N=372** | **High sedentary**  **High PA**  **N=82** | **Low sedentary**  **Low PA**  **N=80** | **Low sedentary**  **High PA**  **N=370** | **P-trend** |
| --- | --- | --- | --- | --- | --- |
| Men  Women | 183 (49%)  189 (51%) | 63 (76%)  19 (24%) | 25 (31%)  55 (68%) | 180 (49%)  190 (51%) |  |
| **Adjusted means** |  |  |  |  |  |
| Age (years) | 72.8 (72.0-73.6) | 69.4 (67.8-71.0) | 72.8 (71.1-74.5) | 69.4 (68.6-70.2) | <0.001 |
| BMI (kg/m^2^) | 23.1 (22.7-23.5) | 22.8 (22.0-23.6) | 22.7 (21.9-23.5) | 22.4 (22.0-22.8) | 0.263 |
| 6 meter walk test (sec) | 7.5 (7.1-7.8) | 6.4 (5.8-7.0) | 7.6 (6.9-8.2) | 7.2 (6.9-7.5) | 0.016 |
| Light-low (%) | 21.6 (21.2-22.1) | 20.7 (19.7-21.6) | 30.7 (29.7-31.6) | 27.6 (27.1-28.0) | <0.001 |

PA: physical activity includes light-high, moderate and vigorous intensity category

*Categorization based on median: sedentary <100 (cpm) <65.4/≥65.4%, Light-high ≥760 (cpm) <9.0/≥9.0% out of total wear time

ANCOVA: analysis of covariance with 95% confidence intervals

Significant P<0.05when no overlap in confidence interval occurs between categories.

Adjusted for sex, education, age, BMI and 6 meter walk test, respectively
